# Biomechanical differences between able-bodied and spinal cord injured individuals walking in an overground robotic exoskeleton

**DOI:** 10.1371/journal.pone.0262915

**Published:** 2022-01-27

**Authors:** Stephen Clive Hayes, Matthew White, Christopher Richard James Wilcox, Hollie Samantha Forbes White, Natalie Vanicek

**Affiliations:** 1 Department of Sport Health and Exercise Sciences, University of Hull, Hull, United Kingdom; 2 Physio Function, Long Buckby, Northamptonshire, United Kingdom; 3 Warwick Medical School, University of Warwick, Warwick, United Kingdom; Toronto Rehabilitation Institute - UHN, CANADA

## Abstract

**Background:**

Robotic assisted gait training (RAGT) uses a powered exoskeleton to support an individual’s body and move their limbs, with the aim of activating latent, pre-existing movement patterns stored in the lower spinal cord called central pattern generators (CPGs) to facilitate stepping. The parameters that directly stimulate the stepping CPGs (hip extension and ipsilateral foot unloading) should be targeted to maximise the rehabilitation benefits of these devices.

**Aim:**

To compare the biomechanical profiles of individuals with a spinal cord injury (SCI) and able-bodied individuals inside the ReWalk^TM^ powered exoskeleton and to contrast the users’ profiles with the exoskeleton.

**Methods:**

Eight able-bodied and four SCI individuals donned a ReWalk^TM^ and walked along a 12-meter walkway, using elbow crutches. Whole-body kinematics of the users and the ReWalk^TM^ were captured, along with GRF and temporal-spatial characteristics. Discreet kinematic values were analysed using a Kruskall-Wallis H and Dunn’s post-hoc analysis. Upper-body differences, GRF and temporal-spatial characteristics were analysed using a Mann-Whitney U test (*P*<0.05).

**Results:**

Walking speed ranged from 0.32–0.39m/s. Hip abduction, peak knee flexion and ankle dorsiflexion for both the SCI and able-bodied groups presented with significant differences to the ReWalk^TM^. The able-bodied group presented significant differences to the ReWalk^TM^ for all kinematic variables except frontal plane hip ROM (*P* = 0.093,δ = -0.56). Sagittal plane pelvic and trunk ROM were significantly greater in the SCI vs. able-bodied (*P* = 0.004,δ = -1; *P* = 0.008,δ = -0.94, respectively). Posterior braking force was significantly greater in the SCI group (*P* = 0.004, δ = -1).

**Discussion:**

The different trunk movements used by the SCI group and the capacity for the users’ joint angles to exceed those of the device suggest that biomechanical profiles varied according to the user group. However, upright stepping with the ReWalk^TM^ device delivered the appropriate afferent stimulus to activate CPGs as there were no differences in key biomechanical parameters between the two user groups.

## Introduction

Spinal cord injury (SCI) at any level can lead to limited function, deficiencies in health and ultimately reduced life satisfaction. Approximately 80% of SCI individuals are wheelchair-dependent for the rest of their life [1]; the wheelchair becomes the platform from which they perform activities of daily living, including home-based and community mobility [2]. Unfortunately various comorbidities are associated with SCI and manual wheelchair use: reduced bone mineral density [3], muscle contractures [4], poor posture and the development of pressure sores [5] are a small sample of SCI sequelae. The impact of some of these conditions can be mitigated through appropriate rehabilitation such as standing [6], stretching [7], strength training [8] and walking [9,10].

Lower limb robotic exoskeletons have been designed as rehabilitative tools and mobility devices, to provide individuals with neuro-muscular deficits a method of upright ambulation. Regular exoskeleton use has the potential to maintain and even improve some of the benefits associated with traditional rehabilitation modalities for a number of SCI-related comorbidities [11]. However, several barriers currently exist related to the practical use of exoskeletons as mobility devices: walking independently can be dangerous for individuals with compromised balance control; the limited speed of walking is prohibitive [12]; as is the requirement to use a walking aid such as elbow crutches, preventing users from carrying anything around the home or work environment [13]. A recent systematic review advocated the use of robotic exoskeletons in SCI rehabilitation as part of a multi-modality approach, with clear recommendations that its use should not be prioritised over other therapies [14].

There is still however a limited understanding of how these devices affect the user’s body, including the impact they have on the central nervous system (CNS). The existence of central pattern generators (CPGs) located in the lower spinal cord in humans is still under debate, with even the definition of CPGs questioned by different groups [15]. There is, however, some evidence to suggest that CPGs may provide a system for regulating cyclic movement which is less susceptible to noise than reflex-based models, and that they may play a role in speed regulation in human gait, through the simplification of complex supraspinal control [15]. If present, the activation of latent CPGs may provide a focus for neurorehabilitation that may facilitate upright stepping following SCI. It has been suggested that, to target potential CPG re-activation in SCI individuals, appropriate afferent feedback in the form of stretch and load sensitive mechanoreceptor in the lower limbs is required [9]. Based on the motor learning principles of repetition, specificity and problem solving [16–18] robotic exoskeletons present an opportunity to facilitate gait re-education and provide the desired afferent feedback to activate CPGs. Previous work has compared exoskeleton walking in the ReWalk^TM^ (ARGO Medical Technologies Ltd., Yokneam, Israel) with speed-matched (SLOW) and preferred speed (NORM) walking in the same group of able-bodied individuals [19]. It was found that the temporal components of exoskeleton gait more closely resembled NORM walking as opposed to SLOW walking whereas the spatial components of exoskeleton gait resembled SLOW gait [19]. The SLOW condition allowed biomechanical differences between able-bodied gait and exoskeleton gait to be identified independent of speed, leading to the conclusion that complex upper body postural control was a significant factor related to balance and continuous stepping in the exoskeleton condition [19]. In “normal” able-bodied gait, maintaining dynamic balance is achieved through the anterio-lateral placement of the swinging limb to the falling centre of mass (COM) [20,21]. During exoskeleton use, this may not be a viable option due to an inability to choose when and where to place the swinging limb; consequently upper body postural adjustments that can alter the direction and acceleration of the falling COM may be a functional alternative, especially when combined with the use of crutches. The programmable design of the ReWalk^TM^ should mean that these findings apply to any ReWalk^TM^ user as long as they can maintain steady gait. However, it is unclear how an SCI user’s body interacts with the device and how this differs from an able-bodied user, especially because the upper body is not controlled by the ReWalk^TM^, meaning that the individual is entirely responsible for upper body postural control.

In order to maximise the potential rehabilitation benefits of overground exoskeletons, the interaction between the user and device needs to be better understood. Therefore, the overarching aim of this study was to assess whether biomechanical differences existed between able-bodied and SCI individuals during overground exoskeleton walking. The first objective was to compare the temporal-spatial characteristics of the two groups. Based on the premise that the ReWalk^TM^ would prescribe the movement to the individual user, and that step length and width were normalised to leg length, it was hypothesised that there would be no significant difference in the temporal-spatial variables between the two groups. The second objective was to identify any differences in range of motion (ROM) and peak joint angles of the lower limbs between the SCI and able-bodied users, and between the ReWalk^TM^ itself and its user. It was hypothesised that the able-bodied users would generate larger ROM and peak angles (in the sagittal plane) than the SCI group, as the SCI individuals do not have the capacity to override the programmed device and would therefore move within the constraints set by the motors. It was however anticipated that able-bodied individuals would generate movements that differed from the angles and ROM generated by the ReWalk^TM^ because they had the neuromuscular capacity to override the exoskeleton device. The third objective was to evaluate upper body movement of the individual, in conjunction with whole-body COM in the vertical and medio-lateral directions, as an indicator of postural control. It was hypothesised that the SCI group would have less COM control than the able-bodied group, resulting in greater trunk excursion angles in the sagittal and frontal planes. The fourth and final objective was to compare the ground reaction forces (GRF) of the two groups. Previous work has demonstrated that the GRFs of able-bodied individuals during exoskeleton gait were significantly lower than NORM walking and that they resembled SLOW gait GRFs [19]. As the walking speed of SCI and able-bodied users should be the same, speed-related differences in GRF profiles were not anticipated.

## Methods

### Participants

Eight able-bodied (mean[SD]: age 28[6] years: height 1.72[0.04] m; mass 77[7] kg) and four complete SCI individuals (age 36[11] years; height 1.81[0.07] m; mass 66[9] kg) were recruited for this study. Healthy able-bodied adults aged 18–60 years, measuring between 160–190 cm in stature, with a mass of less than 100 kg, with no neurological or mobility impairing conditions, and with no musculoskeletal injury were included in the study. Individuals with an SCI were included if they met the same inclusion criteria except for having a lesion to their spinal cord. Spinal cord injured participants were also required to be motor-complete (ASIA A-B) injury level of T2 or below, and must have been classified as an experienced ReWalk^TM^ user (defined as a user capable of completing the basic skill assessment established by ReWalk^TM^ [22] with a minimum of 20 hours’ previous use). Participant demographics, including injury level and ASIA score, are presented in Table 1. Other pre-requisites for the safe use of the ReWalk^TM^ included the ability to transfer independently between two stable level surfaces, arm use and some hand function, and the capacity to tolerate upright positioning for a minimum of 30 minutes without experiencing light headedness or a drop in blood pressure. Ethical approval was provided by the University of Hull’s departmental review board (reference number 1415213). All participants gave their written informed consent prior to testing.

**Table 1:** Participant characteristics.

Participant Number	Designation	Age	Gender	Height (cm)	Body Mass (kg)	Injury Level	Training hours
P001	AB	30	Male	174.1	90.2	N/A	1
P002	AB	26	Male	169.0	77.8	N/A	1
P003	AB	28	Male	170.5	67.5	N/A	1
P004	AB	23	Female	165.0	71.5	N/A	1
P005	AB	23	Female	177.2	77.9	N/A	1
P006	AB	42	Male	177.8	77.4	N/A	1
P007	AB	26	Male	171.4	72.6	N/A	1
P008	AB	24	Female	172.1	77.9	N/A	1
**Mean (SD)**		**27.75 (6.25)**		**172.13 (4.24)**	**76.6(6.7)**		**1**
P009	SCI	40	Female	177.8	55.0	ASIA A (T9)	20+
P010	SCI	50	Male	175.0	77.0	ASIA B (T4)	20+
P011	SCI	21	Male	178.0	60.1	ASIA B (T10)	20+
P012	SCI	32	Male	193.0	71.5	ASIA A (T5)	20+
**Mean (SD)**		**35.75 (10.64)**		**180.95 (7.06)**	**65.9 (8.8)**		**20+**

### Protocol

Testing consisted of a single visit to the University Human Performance laboratory for the SCI participants and two visits for the able-bodied participants; one ReWalk^TM^ training session and a testing session. All able-bodied participants were instructed in the use of the exoskeleton by a ReWalk^TM^ trainer and physiotherapist, all participants were capable of ambulating in the ReWalk^TM^ with minimal assistance from the trainer after one hour of training. All participants were fitted for the ReWalk^TM^ upon arrival. Standardised settings according to manufacturer specifications were programmed for all participants. Pre-programmed peak angles in the sagittal plane were set as follows in this study: hips (extension 8^o^, flexion 22^o^) and knees (flexion 46^o^). The spring loaded mechanism used by the ReWalk^TM^ has a maximum dorsiflexion angle of 10^o^, this mechanism was locked at maximum dorsiflexion. Step latency was set at 0 ms, step duration was set at 700 ms and the pelvic tilt delta angle (for the sensor fixed on the pelvic bracket to initiate stepping) was set at 7^o^ anterior tilt [22]. All participants ambulated with forearm crutches for balance and were followed closely by a certified ReWalk^TM^ trainer during exoskeleon use. All participants were given a 30-minute re-familiarisation session in the ReWalk^TM^ prior to preparation for the testing session.

Participants wore form-fitting clothing for the testing and were provided with standardised trainers that fit their feet and the ReWalk^TM^ footplate. A total of 105 retro-reflective markers (14 mm) were used to track the motion of the user and the ReWalk^TM^, 73 of these markers were used to track the body and the remaining 32 were used to track the ReWalk^TM^ and crutches. Body segments and ReWalk^TM^ segments were defined by an end point or joint centre based upon anatomical locations or ReWalk^TM^ technical specifications and the calibrated anatomical systems technique [23]. Tracking marker clusters were affixed to each body segment and each ReWalk^TM^ segment simultaneously. Each segment was tracked using the six-degrees-of-freedom principles [24]. Three-dimensional kinematics were captured with ten Oqus 4.0 cameras (Qualisys, Gothenburg, Sweden) at 100 Hz and synchronised with two floor integrated Kistler (9286AA) force plates (Winterthur, Switzerland) sampling at 1000 Hz via Qualisys Track Manager software version 2.15 (Gothenburg, Sweden).

Participants were asked to walk along a five-meter walkway ten times. They were not given any instructions about walking speed, which should have been determined by the ReWalk^TM^ settings. As the step latency, step duration and the available ROM at the hips were the same for all participants, step length (based on the leg length of each individual) should have been the predominant factor influencing speed. This suggests that speed would be slightly different for each participant. The starting point of each walking trial was determined *a priori* to facilitate GRF data collection. This was because at least one step with each foot was required before and after contact with the force plates to ensure the data analysed were not representative of gait initiation or termination. Kinetic data were discarded if a complete foot contact was not made with the force plate.

### Data reduction

3D marker coordinate and GRF data were processed in Visual 3D Version 5 (C-Motion, MD, USA). Kinematic data were interpolated using a third order polynomial. Kinematic and kinetic data were low-pass filtered using fourth order Butterworth filters (cut-off 6 Hz and 30 Hz, respectively). Joint kinetics were not calculated as the lower limb joints were robotically assisted by the ReWalk^TM^. All variables were time normalised to the gait cycle starting with initial contact; GRFs were normalised to combined body and ReWalk^TM^ mass. The vertical GRF peaks, defined as vertical loading and vertical push-off, were identified based on percentage gait cycle. The following kinematics were identified for all users and the ReWalk^TM^: ankle, knee and hip peak joint angles and ROM (degrees) and peak frontal plane hip angles. These variables were averaged across both the right and left limbs for each individual. Trunk and pelvis segment excursions (degrees) were reported in all three planes for the user only. Peak vertical and anterior-posterior GRFs (N/kg) were compared between groups. Centre of mass medio-lateral and vertical displacements were normalised to body height (%). Medio-lateral COM was offset using a Euclidean distance correction factor as individuals did not walk along the x-axis of the laboratory co-ordinate system. The Euclidian distance correction factor was calculated by identifying the mean of all data points in the medio-lateral COM and subtracting this value from each data point.

### Statistical analysis

All data were analysed using SPSS statistical package (V22, IBM statistics, Armonk, NY). Lower limb kinematic data were analysed using a Kruskall-Wallis H and Dunn’s post-hoc analysis [25]. The distribution shapes were not similar for any of the 12 variables, as such interpretations were based on mean rank scores. The remaining data were all analysed using a Mann-Whitney U test, distribution shapes were not similar for any variables, mean rank scores were again used. Statistical significance was set at *P* < 0.05. Non-parametric Cliff’s Delta effect sizes were calculated [26]. Established thresholds of small (0.147–0.33) medium (0.33–0.474) and large (>0.474) were used for interpretation [27].

## Results

### Temporal-spatial characteristics

Temporal-spatial parameters for able-bodied and SCI REWalk^TM^ gait are displayed in Table 2. Significant reductions in step length and cadence (*P* = 0.004 and *P* = 0.028, respectively) resulted in a significantly slower walking speed for the SCI group (*P* = 0.016, δ = 0.88, 95% CI 0.99 to 0.19), leading to a potentially meaningful increase in time spent in double support (δ = -0.56, 95% CI -0.94 to 0.41).

**Table 2:** Median (IQR) temporal-spatial data for able-bodied and SCI ReWalk^TM^ gait. (Mann Whitney U tests, significance set at 95%, Cliff’s delta effect sizes and 95% confidence intervals).

Temporal-spatial parameters	AB Medians (IQR)		SCI Medians (IQR)		AB Mean Rank	SCI Mean Rank	U	Sig (*p*)	Effect size δ (95% CI)			
Walking speed (m/s)	0.39	(0.04)	0.32	(0.03)	8.25	3.00	2.00	0.016*	0.88	(0.99	to	0.19)
Double support time (%)	26	(4.6)	34	(5.5)	5.31	8.88	25.50	0.109	-0.56	(0.41	to	-0.94)
Cadence (steps/min)	48	(2)	46	(2)	8.13	3.25	3.00	0.028*	0.88	(0.99	to	0.19)
Stance time (%)	64	(2.0)	68	(2.6)	5.13	9.25	27.00	0.730	-0.69	(0.21	to	-0.96)
Swing time (%)	37	(2.3)	33	(2.7)	7.63	4.25	7.00	0.154	0.56	(0.94	to	-0.41)
Step length (% leg length)	52	(8.1)	45	(1.5)	8.50	2.50	0.00	0.004*	1			
Step width (% leg length)	18	(2.6)	15	(1.2)	8.50	2.50	0.00	0.004*	1			

Comparison of mean ranks, distribution shapes not similar.

AB = Able-bodied, SCI = Spinal cord injured, IQR = interquartile range, Sig = Significance, U = Mann Whitney U statistic, GRF = Ground reaction force.

* = significant difference, alpha level 0.05.

Using trigonometry based on the hip flexion angle of 22^o^ and a hip extension angle of 8^o^ (total of 30^o^), step length should be 52% leg length for every participant. The median step length of able-bodied individuals was 52% leg length, however in the SCI group the median step length was 45% leg length (Table 2) which was significantly shorter (*P* = 0.004).

### Lower limb kinematics

**Fig 1:**
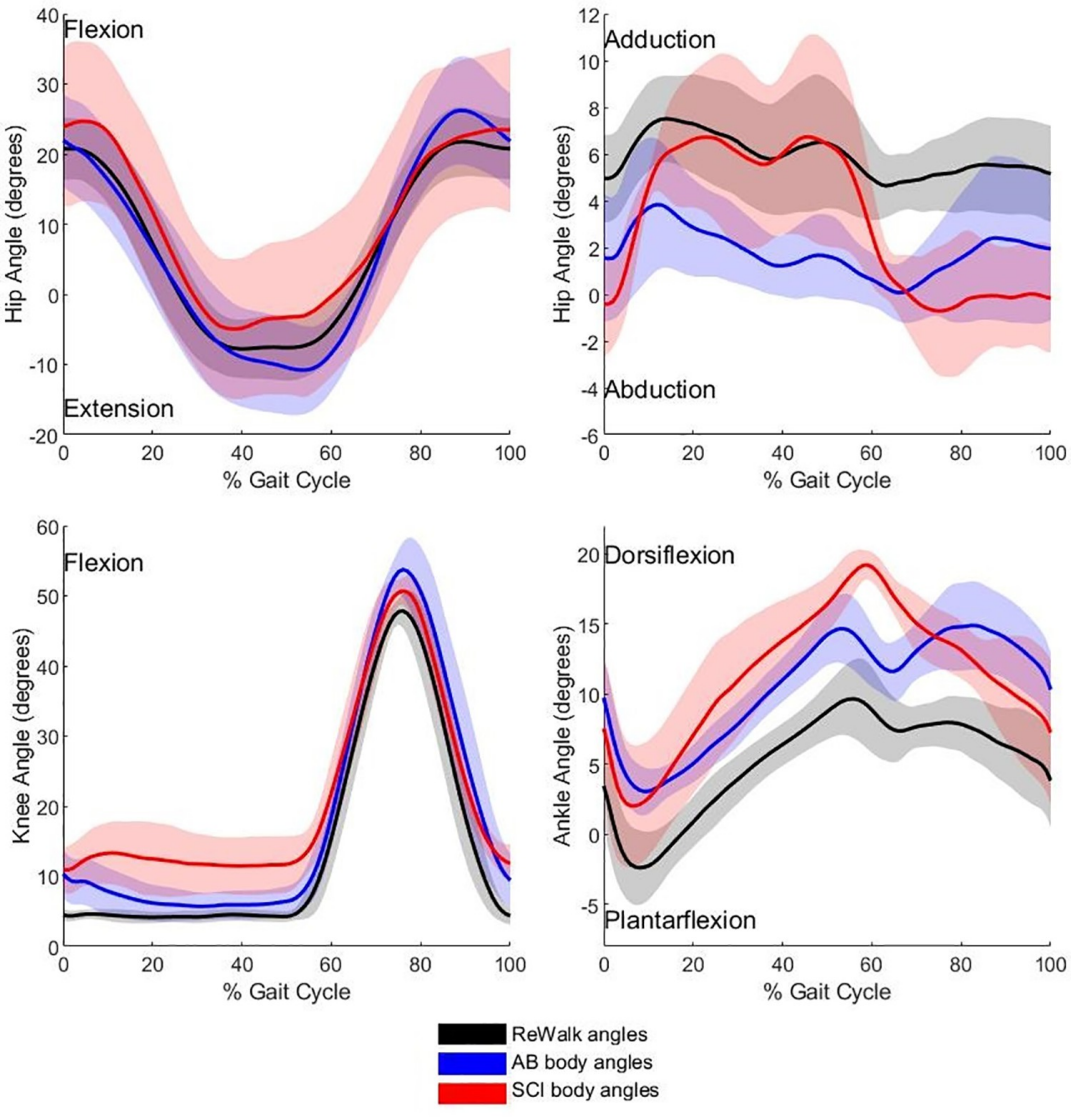
Sagittal and frontal plane hip and sagittal plane knee and ankle joint angles of the ReWalk^TM^, able-bodied and SCI individuals. Data averaged across both limbs. The gait cycle commences and terminates with ipsilateral foot contact. Flexion, dorsiflexion and adduction are positive.

Lower limb peak kinematic and ROM data are presented in Fig 1 and Table 3. Twelve lower limb variables were analysed for the hip, knee and ankle joints. Significant differences were identified for nine of the twelve variables. Results from the post-hoc analyses revealed that the greatest differences in peak joint angles and ROM existed between the able-bodied users and the ReWalk^TM^. Furthermore, large effect sizes were evident for all variables between the two groups. The frontal hip excursion, and sagittal knee flexion and ankle dorsiflexion during terminal stance, for both the SCI and able-bodied groups, all presented with significant differences to the ReWalk^TM^. During frontal plane hip motion, the ReWalk^TM^ limb maintained an adducted position throughout the gait cycle, whereas the SCI and able-bodied users’ hips abducted beyond neutral to ~1.5^o^. Able-bodied and SCI knee flexion during swing (SCI mean rank = 17.25, AB mean rank = 19.13, RW mean rank = 6.50, *P* = 0.025 and *P* > 0.001) and ankle dorsiflexion (SCI mean rank = 22.00, AB mean rank = 16.25, RW mean rank = 6.83, *P* = 0.001 and *P* = 0.011) were both significantly greater than the peak angles generated by the ReWalk^TM^. However, knee ROM was the only variable that was significantly different (~5.5^o^) between the able-bodied and SCI groups.

**Table 3:** Median (IQR) lower limb peak kinematic values and joint range of motion (ROM) for able-bodied and SCI ReWalk^TM^ gait (degrees). (Kruskall-Wallis H test, significance set at 95%, Dunn’s post-hoc test and Cliff’s delta effect sizes and 95% confidence intervals).

	Median AB Angles (IQR)		Median SCI Angles (IQR)		Median RW Angles (IQR)		Mean Rank			Χ^2^(2)	Sig (*p*)	Effect Size
							AB	SCI	RW			
Hip ROM (sagittal)	38.7	(2.1)	32.6	(4.4)	31.1	(2.0)	20.13	11.50	7.75	14.797	0.001*	0.64
Hip ROM (frontal)	5.7	(0.5)	8.3	(8.6)	4.1	(1.4)	15.88	16.50	8.92	6.184	0.045*	0.27
Hip flexion swing	28.0	(10.3)	30.4	(8.3)	21.6	(4.7)	14.88	15.25	10.00	3.007	0.222	0.13
Hip extension stance	-9.2	(9.1)	-1.4	(7.3)	-10.3	(4.6)	10.75	17.00	12.17	2.137	0.344	0.09
Hip adduction stance	5.0	(1.8)	7.2	(4.7)	7.8	(1.1)	6.75	14.75	15.58	7.977	0.019*	0.35
Hip abduction swing	-1.0	(2.5)	-1.5	(3.4)	3.7	(1.6)	8.25	7.00	17.17	10.537	0.005*	0.46
Knee ROM (sagittal)	51.9	(3.3)	46.4	(3.6)	47.4	(0.7)	20.50	6.25	9.25	15.9	0.000*	0.69
Knee flexion LR	7.8	(1.9)	11.9	(3.0)	4.8	(1.3)	15.75	21.75	7.25	15.15	0.001*	0.66
Knee flexion swing	54.9	(3.7)	54.6	(2.2)	50.4	(0.9)	19.13	17.25	6.50	17.468	0.000*	0.76
Ankle ROM (sagittal)	13.6	(2.4)	18.4	(5.3)	12.8	(3.8)	12.38	18.00	10.75	3.158	0.206	0.14
Ankle dorsiflexion TS	14.5	(3.5)	20.6	(0.7)	10.0	(3.3)	16.25	22.00	6.83	17.177	0.000*	0.75
Ankle plantarflexion swing	1.6	(2.4)	1.3	(7.0)	-2.4	(3.7)	18.63	15.25	7.50	12.607	0.002*	0.55
Post-Hoc Analysis	RW vs. SCI				RW vs. AB				SCI vs. AB			
	Mean Rank Difference	Sig (*p*)	Effect Size δ		Mean Rank Difference	Sig (*p*)	Effect Size δ		Mean Rank Difference	Sig (*p*)	Effect Size δ	
Hip ROM (sagittal)	3.75	1.000	-0.38 (0.40 to -0.84)		12.38	0.000*	-1		8.63	0.139	-1	
Hip ROM (frontal)	7.58	0.190	-0.67 (0.02 to -0.93)		6.96	0.093	-0.56 (0.01 to -0.86)		-0.63	1.000	0.00 (0.75 to -0.75)	
Hip adduction stance	0.83	1.000	0.00 (0.70 to -0.70)		8.83	0.019*	0.77 (0.95 to 0.23)		8.00	0.194	0.56 (0.91 to -0.25)	
Hip abduction swing	10.17	0.038*	0.83 (0.97 to 0.24)		8.92	0.017*	0.75 (0.94 to 0.22)		1.25	1.000	-0.13 (0.55 to -0.70)	
Knee ROM (sagittal)	3.00	1.000	0.38 (0.87 to -0.51)		11.25	0.001*	-1		14.25	0.003*	-1	
Knee flexion LR	14.50	0.001	-1		8.50	0.025*	-0.81 (-0.34 to -0.96)		6.00	0.498	0.81 (0.97 to 0.09)	
Knee flexion swing	10.75	0.025*	-1		12.63	0.000*	-1		1.88	1.000	-0.31 (0.41 to -0.79)	
Ankle dorsiflexion TS	15.17	0.001*	-1		9.42	0.011*	-0.81 (-0.37 to -0.95)		5.75	0.553	0.88 (0.99 to 0.19)	
Ankle plantarflexion swing	7.75	0.173	-0.50 (0.28 to -0.88)		11.13	0.002*	-1		3.38	1.000	-0.06 (0.71 to -0.77)	

AB = Able-bodied, SCI = Spinal cord injured, RW = ReWalk^TM^, IQR = interquartile range TS = Terminal stance, LR = Loading response, Sig = Significance, X^2^(2) = Chi-squared statistic (degrees of freedom).

* = significant difference, alpha level 0.05.

### Centre of mass and postural control

**Fig 2:**
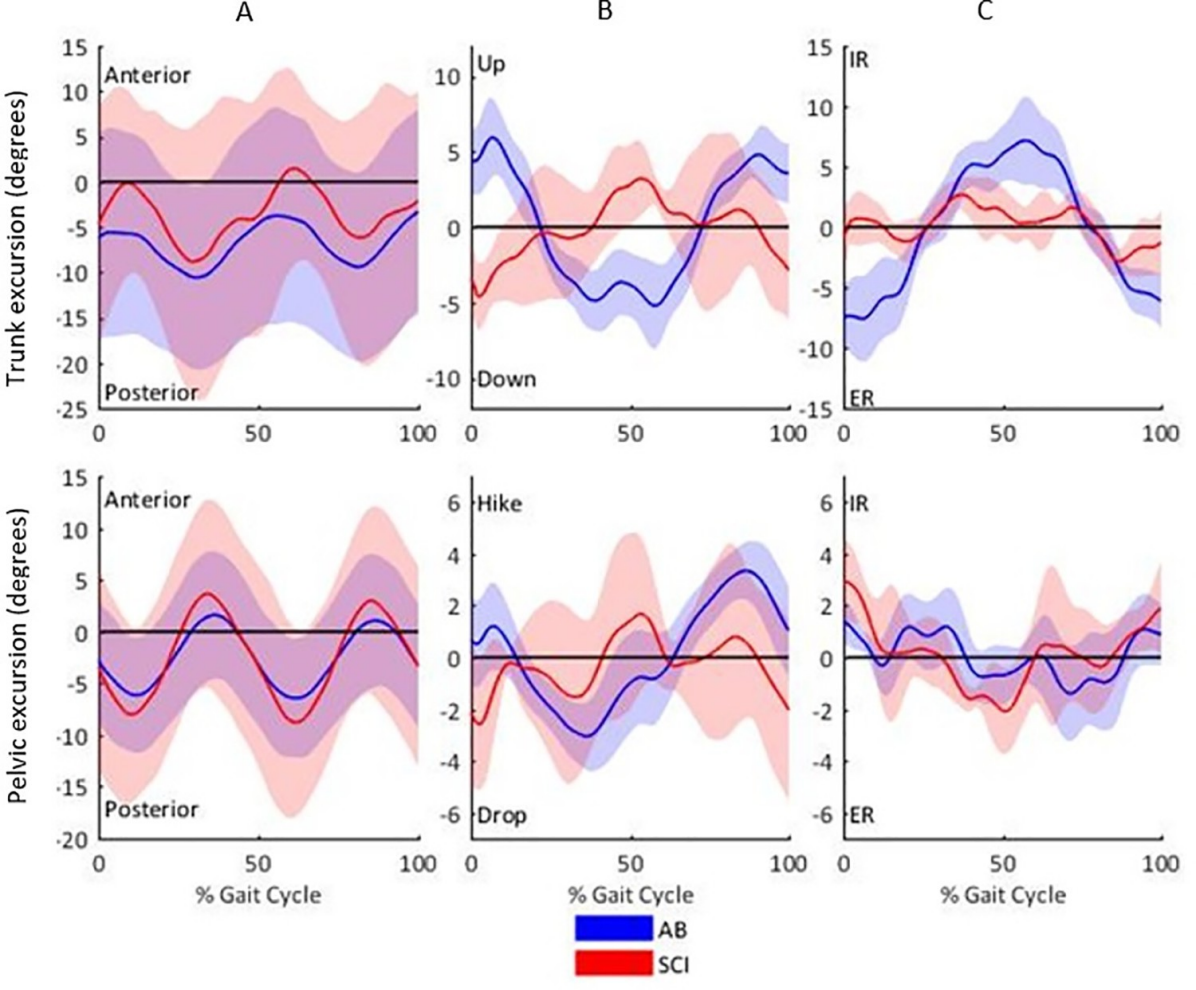
Trunk and pelvis segment excursions in the (A) sagittal, (B) frontal and (C) transverse planes for able-bodied and SCI individuals. The gait cycle commences and terminates with ipsilateral foot contact. Internal rotation is transverse rotation in an anterior direction with the side of the body defined by the lead limb at commencement of the gait cycle.

Trunk and pelvic ROM and peak segment excursions are reported in Table 4 and displayed in Fig 2. Sagittal ROM for the trunk was ~8.6^o^ greater in SCI individuals (able-bodied mean rank = 4.63, SCI mean rank = 10.25, U = 37.00, *P* = 0.008) and the pelvic sagittal ROM was ~5.4^o^ greater in SCI individuals (able-bodied mean rank = 4.50, SCI mean rank = 10.50, U = 36.00, *P* = 0.004) compared to the able-bodied group. Although no significant differences were identified in the frontal and transverse planes for either the trunk or pelvic kinematics, the waveforms presented in Fig 2 show opposing movement patterns between the two groups.

**Table 4:** Median (IQR) trunk and pelvis kinematic values and joint range of motion (ROM) for able-bodied and SCI ReWalk^TM^ gait (degrees). (Mann Whitney U tests, significance set at 95%, Cliff’s delta effect sizes and 95% confidence intervals).

Upper body kinematics	AB Median Angle (IQR)		SCI Median Angle (IQR)		AB Mean Rank	SCI Mean Rank	U	Sig (*p*)	Effect size δ (95% CI)			
Trunk ROM (sagittal)	12.6	(2.1)	21.2	(4.1)	4.63	10.25	37.00	0.008*	-0.94	(-0.36	to	-0.10)
Trunk ROM (frontal)	14.5	(6.4)	16.5	(6.8)	6.13	7.25	49.00	0.683	-0.19	(0.55	to	-0.76)
Trunk ROM (transverse)	21.1	(8.0)	16.6	(9.3)	7.38	4.75	19.00	0.283	0.44	(0.85	to	-0.31)
Pelvis ROM (sagittal)	11.0	(2.6)	16.4	(1.8)	4.50	10.50	36.00	0.004*	-1			
Pelvis ROM (frontal)	8.0	(1.8)	11.0	(4.2)	5.50	8.50	44.00	0.214	-0.50	(0.35	to	-0.90)
Pelvis ROM (transverse)	7.9	(1.9)	9.0	(1.8)	5.63	8.25	45.00	0.283	-0.44	(0.32	to	-0.85)

Comparison of mean ranks, distribution shapes not similar.

AB = Able-bodied, SCI = Spinal cord injured, IQR = interquartile range, Sig = Significance, U = Mann Whitney U statistic, COM = Centre of mass.

* = significant difference, alpha level 0.05.

**Fig 3:**
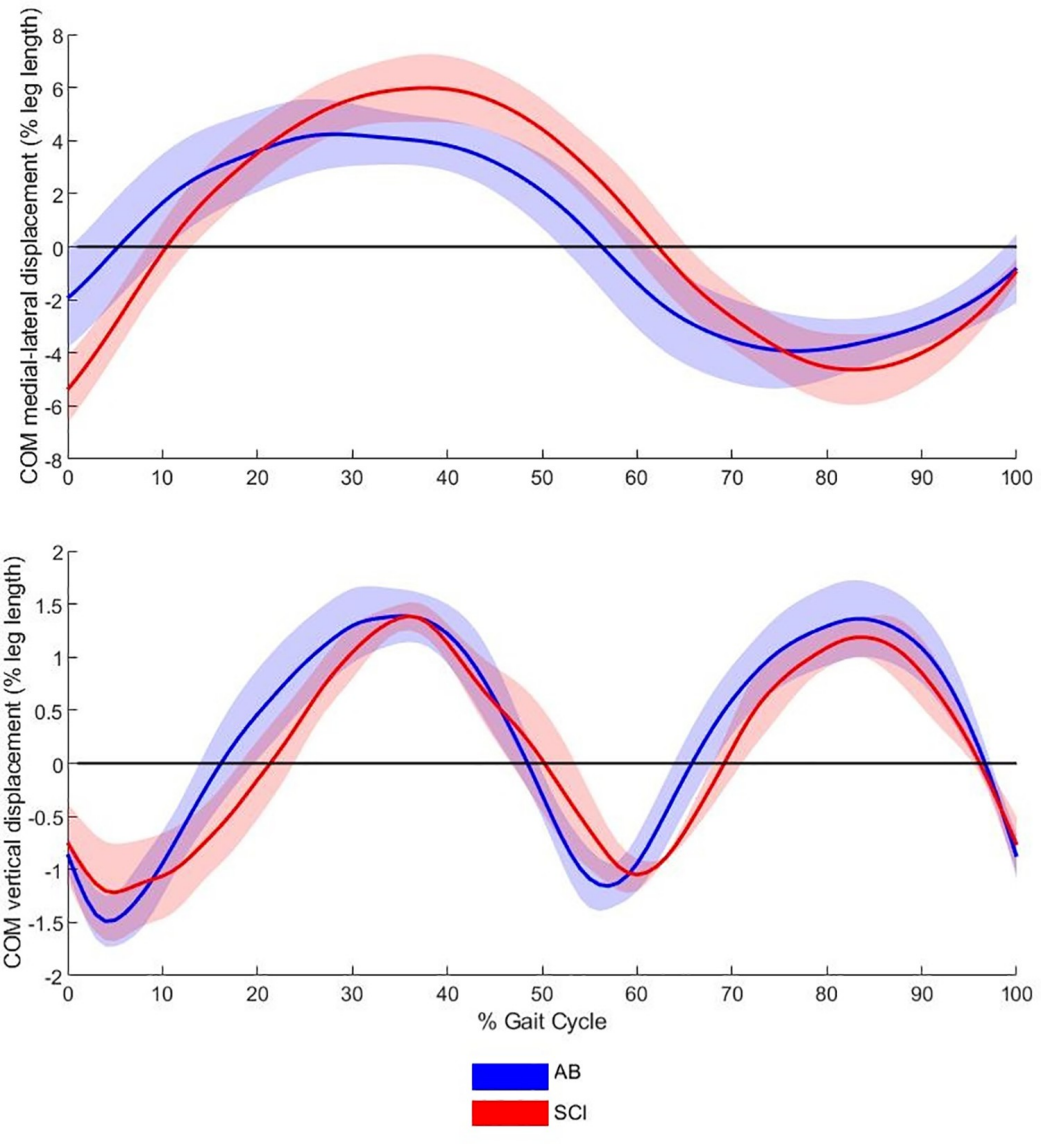
Centre of mass displacement (% leg length) of able-bodied and SCI individuals in the (A) medial-lateral and (B) vertical directions. Medial-lateral displacement data were exposed to a Euclidian correction factor to facilitate a change of sign as movement direction changed. The gait cycle commences and terminates with ipsilateral foot contact. IR = internal rotation and ER = external rotation.

Frontal and vertical centre of mass displacement data are presented in Table 5 and Fig 3. Although no significant differences were identified for COM displacement, the medio-lateral COM displacement variables all presented with large effect sizes (δ = > 0.474) suggesting that the medio-lateral COM movement may have been greater for SCI individuals. This can be seen more clearly in Fig 3A.

**Table 5:** Median (IQR) centre of mass displacement values for able-bodied and SCI ReWalk^TM^ gait (% leg length). (Mann Whitney U tests, significance set at 95%, Cliff’s delta effect sizes and 95% confidence intervals).

Centre of mass displacement	AB Median Displacement (IQR)		SCI Median Displacement (IQR)		AB Mean Rank	SCI Mean Rank	U	Sig (*p*)	Effect size δ (95% CI)			
COM medial-lateral max	4.75	(0.71)	5.27	(1.68)	5.38	8.75	25.00	0.154	-0.56	(0.18	to	-0.90)
COM medial-lateral min	-4.59	(0.74)	-5.77	(1.62)	7.75	4.00	6.00	0.109	0.63	(0.92	to	-0.14)
COM medial-lateral range	9.34	(1.44)	11.49	(3.30)	5.25	9.00	26.00	0.109	-0.63	(0.14	to	-0.92)
COM vertical max	1.56	(0.17)	1.44	(0.16)	7.25	5.00	10.00	0.154	0.38	(0.82	to	-0.35)
COM vertical min	-1.53	(0.31)	-1.38	(0.34)	5.88	7.75	21.00	0.214	-0.31	(0.41	to	-0.79)
COM vertical range	3.07	(0.46)	2.80	(0.52)	7.25	5.00	10.00	0.368	0.38	(0.82	to	-0.35)

Comparison of mean ranks, distribution shapes not similar.

AB = Able-bodied, SCI = Spinal cord injured, IQR = interquartile range, Sig = Significance, U = Mann Whitney U statistic, COM = Centre of mass, Max = Maximum Min = Minimum.

* = significant difference, alpha level 0.05.

### Ground reaction forces

**Fig 4:**
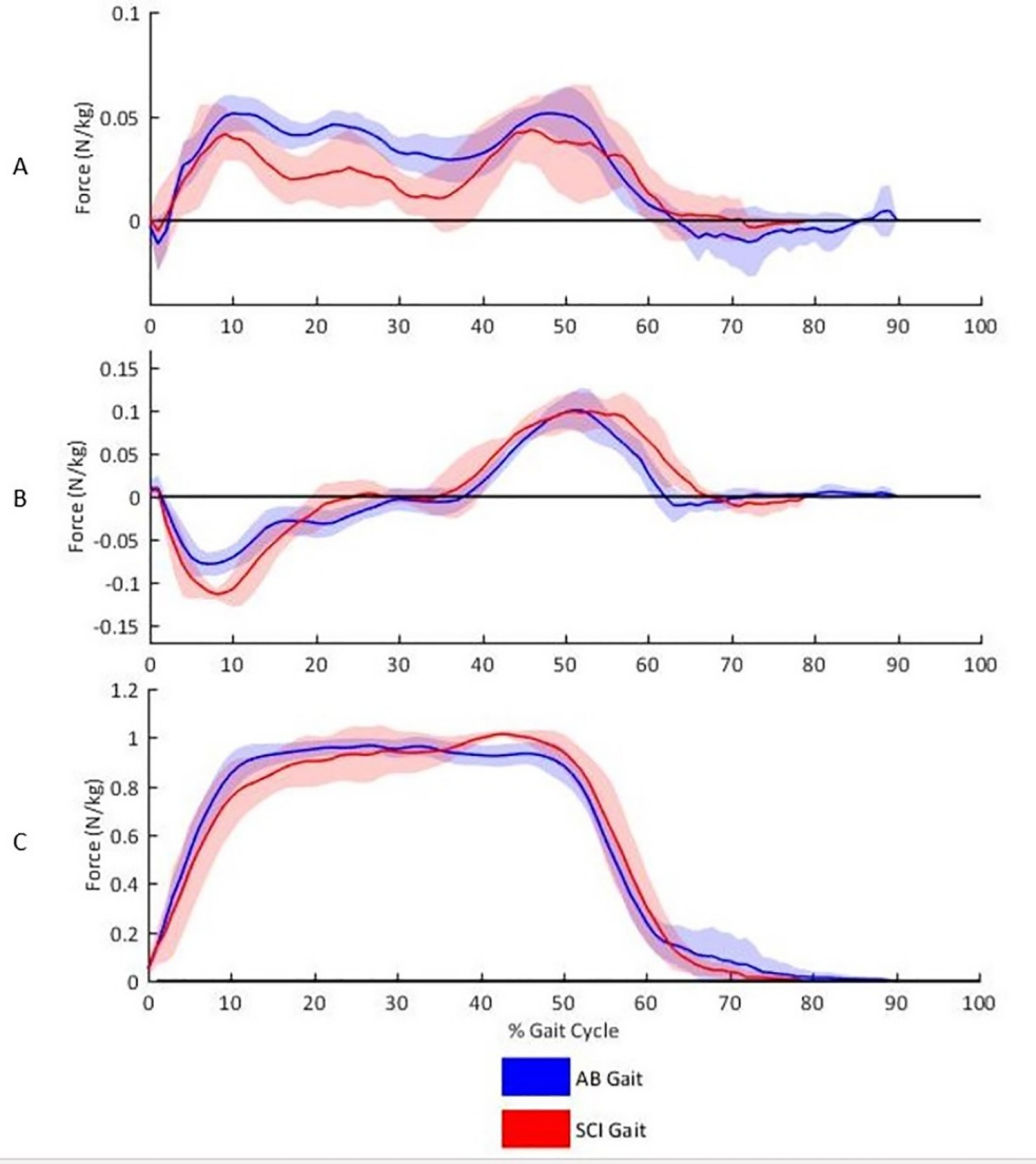
(A) Medial-lateral, (B) anterior-posterior and (C) vertical ground reaction forces of able-bodied and SCI individuals. Data were normalised to body mass and ReWalk^TM^ mass (N/kg). The gait cycle commences and terminates with ipsilateral foot contact. (A) Lateral and (B) anterior propulsion forces are positive.

Fig 4 illustrates the GRF profiles of able-bodied and SCI ReWalk^TM^ gait. Significantly greater forces were identified for the SCI group in loading in the anterior-posterior direction (able-bodied mean rank = 8.60, SCI mean rank = 2.50, U = 0.00, *P* = 0.004) and in preparation for toe off (~45% of gait cycle) in the vertical direction (able-bodied mean rank = 4.62, SCI mean rank = 10.25, U = 31.00, *P* = 0.008). Based on the large effect size, able-bodied individuals presented with a greater load rate but this was not significantly different (δ = 0.56, 95% CI: 0.91 to -0.25) (Table 6).

**Table 6:** Median (IQR) peak ground reaction forces (N/kg) and load/decay rates (N/kg/s) for able-bodied and SCI ReWalk^TM^ gait. (Mann Whitney U tests, significance set at 95%, Cliff’s delta effect sizes and 95% confidence intervals).

Ground reaction forces	AB Median GRF (IQR)		SCI Median GRF (IQR)		AB Mean Rank	SCI Mean Rank	U	Sig (*p*)	Effect Sizes δ (95% CI)			
Lateral	0.07	(0.01)	0.07	(0.02)	6.38	6.75	17.00	1.000	0.06	(0.64	to	-0.71)
Anterior propulsion	0.13	(0.02)	0.14	(0.01)	6.12	7.25	19.00	0.683	-0.19	(0.47	to	-0.71)
Posterior braking	-0.10	(0.01)	-0.15	(0.02)	8.60	2.50	0.00	0.004*	-1			
Vertical loading	1.01	(0.02)	0.96	(0.16)	6.88	5.75	13.00	0.683	0.19	(0.80	to	-0.62)
Minimum vertical force in stance	0.87	(0.05)	0.83	(0.10)	7.25	5.00	10.00	0.368	0.38	(0.84	to	-0.40)
Vertical push-off	1.03	(0.03)	1.09	(0.04)	4.62	10.25	31.00	0.008*	-0.94	(-0.36	to	-1.00)
Load rate (N/kg/s)	1.39	(0.17)	1.21	(0.34)	7.63	4.25	7.00	0.154	0.56	(0.91	to	-0.25)
Decay rate (N/kg/s)	-1.44	(0.39)	-1.35	(0.28)	7.25	5.00	10.00	0.368	0.38	(0.84	to	-0.40)

Comparison of mean ranks, distribution shapes not similar.

AB = Able-bodied, SCI = Spinal cord injured, IQR = interquartile range, Sig = Significance, U = Mann Whitney U statistic, GRF = Ground reaction force.

* = significant difference, alpha level 0.05.

## Discussion

The aim of the current study was to determine if there were any biomechanical differences between able-bodied and SCI ReWalk^TM^ users during overground walking. It was hypothesised that there would be no significant differences between groups relating to the temporal-spatial gait parameters because of the programmable nature of exoskeleton gait. Yet several significant differences were identified: reduced step length and cadence in the SCI group, contributing to their slower walking speeds. Whole-body movements should be considered to understand why these differences existed. The timing and magnitude of trunk movement during ReWalk^TM^ use appeared to explain several biomechanical differences between able-bodied and SCI users. Specifically, the anterior orientation and timing of trunk movement influenced step length, posterior (braking) GRF and subsequently walking speed.

Sagittal plane ROM of the trunk (Table 4) was significantly greater in SCI individuals and although the trunk maintained a predominantly posterior orientation for both groups, the SCI group also displayed a more anterior position throughout the gait cycle (Fig 2). If the user’s trunk rotated anteriorly too early or too far during swing this could lead to an anterior rotation of the whole body and insufficient ground clearance, leading to early ipsilateral contact and a shorter step length. In this instance, the power of the motor would no longer drive the swinging limb forward but would push the rest of the body backwards. This could also account for the significantly larger posterior GRF in the SCI group (Table 6). Combined with the significant reduction in cadence of two steps per minute, these variables explain the significantly slower walking speed in the SCI group. To the best of our knowledge, this is the first study to incorporate anterior-posterior GRF data for individuals with an SCI during exoskeleton walking. Therefore no previous work has been able to identify the link between reduced speed and reduced step length yet increased braking force during overground walking with an exoskeleton.

As the ReWalk^TM^ settings were identical for both groups, any temporal variation was ultimately a product of the user orientating the body differently to initiate step transition. During ReWalk^TM^ gait, step transition has to be triggered. Activation of step transition occurs through the orientation of a tilt sensor located on the left lateral portion of the pelvic bracket [28]. The temporal differences between groups may be explained by differences in the time taken to orientate the body between steps. Although both groups needed to maintain postural control, the able-bodied individuals could utilise their core muscles and neuromuscular feedback to achieve the specific body orientation to facilitate step transition more quickly, with greater positional control and a reduced trunk ROM compared to the SCI group (median difference -8.6^o^, *P* = 0.008). Therefore, it can be assumed that the temporal variances leading to reduced cadence in the SCI group were independent of the ReWalk^TM^ and were due to how the individual was able to orientate themselves and how quickly they could achieve this.

It was hypothesised that SCI users would experience smaller lower limb ROM and reduced peak angles than able-bodied users whilst generating larger upper body ROM within the ReWalk^TM^. Table 3 shows that significant differences were identified between the able-bodied and SCI individuals and the ReWalk^TM^ at the hip, knee and ankle joints. Post-hoc analysis revealed that the decrease in sagittal knee ROM for SCI users was the only significant difference between able-bodied and SCI users. Fig 1 illustrates that the knee joint in the SCI group experienced an increased level of flexion throughout stance. Practically, this was likely caused by the strapping system used to hold the user’s limbs against the ReWalk^TM^. The flexible webbing allowed the individual to drop (‘sag’) within the device, pushing the knee into approximately 10^o^ of flexion. This was evident in the SCI group as they lacked the neuromuscular control to counteract this ‘sagging’. Although not directly measured this would have changed the alignment of the individuals’ knee with the ReWalk^TM^ knee joint centre, which may also have impacted the alignment of the hip joints. Newer versions of the ReWalk^TM^ have subsequently installed a physical stop immediately inferior to the knee to prevent the anterior drop of the tibia. The capacity of any robot assisted gait training device to deliver prescribed movement patterns is based on the alignment of joint centres [29]. However Hidler et al. [29] identified joint misalignment in able-bodied individuals walking in the Lokomat® (Hocoma, AG, Volketswill, Switzerland) and they concluded that the misalignment provided the capacity for variability within the gait cycle. The principles of motor learning include task specificity, training intensity and task variability [9]. Task variability has positive implications for learning (or re-learning) tasks [30] and can prevent the spinal cord from entering a state of learned helplessness, where a lack of capacity for exploration will result in poor skill acquisition [31].

The concept of variability in motor learning and the triggering of locomotor CPGs has previously been explored with the negative implications of the patient not actively engaging with the movement and therefore remaining passive [32–34]. Although joint centre misalignment was likely in both groups, the reasoning for each was very different. The able-bodied group most likely demonstrated variability equivalent to and for the same reasons as those identified by Hidler et al. [29] as they tried to operate beyond the ReWalk’s^TM^ mechanical constraints. As discussed, the SCI individuals appeared to have dropped within the device altering their position relative to the ReWalk^TM^, leading to a limited yet, persistent change in the user kinematics. Three variables presented with significant differences between the SCI group and the ReWalk^TM^ itself, compared to eight of nine significant kinematic variables between the able-bodied group and the ReWalk^TM^. This suggests that the SCI users more closely followed the movements of the ReWalk^TM^ compared to the able-bodied group. The active involvement of the trunk to maintain balance and facilitate stepping may also provide sufficient engagement in the activity to prevent participant passivity.

In order to activate the CPGs and elicit a stepping response, appropriate afferent feedback must be sent from the muscle spindles of the hip flexors coupled with unloading of the ipsilateral foot. The average sagittal hip ROM for the able-bodied and the SCI groups were equivalent to the previously explored slow walking gait data presented in Hayes et al. [19]. Coupled with the vertical GRF data presented in Table 6, showing equal unloading between the two groups, there is an argument to suggest that the afferent stimulation at the hip joint, and ipsilateral lower limb unloading, may still provide sufficient stimulus to trigger stepping CPGs [30]. This is based on the premise that once a step has been initiated through activation of the tilt sensor, the motor at the hip joint of the ReWalk^TM^ facilitates hip flexion of the trail leg. The subsequent change in hip position, its effect on the mechanoreceptors in the hip flexors and the change in trail leg loading, would represent appropriate afferent input to potentially activate the latent CPGs, providing a targeted neuro-rehabilitative stimulus [9,15].

It was hypothesised that the SCI group would exhibit a greater displacement of whole-body COM in the medial lateral and vertical directions during exoskeleton overground walking than the able-bodied group. The vertical component of the COM displayed no significant differences and small to moderate effect sizes (δ = -0.31 to 0.38) between the groups. Although discreet values of lateral trunk displacement did not show any significant differences between the groups, the waveforms presented in Fig 2 show that the two groups generated opposing movement patterns, suggesting that the frontal plane movement control differed between the groups. There were no significant differences between the groups for the medial-lateral COM displacement, however large effect sizes were evident (max δ = -0.56, min δ = 0.63 and range δ = -0.63) potentially suggesting greater medio-lateral displacement in the able-bodied group, in contrast to the hypothesis. Fig 3 demonstrates that the peak lateral displacements of the COM occurred at the same time as the maxima of the vertical displacement of COM in both groups and that these presented with the same waveform for both groups. In normal walking, the lateral position of the swinging limb’s ground contact (i.e., step width) relative to the falling COM influences frontal plane stability [35]. To maintain frontal stability, the ReWalk^TM^ could generate a larger step width to produce a sufficiently wide base of support within which the COM could be maintained [36]. The lack of robotic articulation in the frontal plane means that step width was predominantly controlled by the size of the pelvic bracket and the rigidity of the exoskeleton. Able-bodied individuals who presented with a significantly greater step width (*p* = 0.004) would have been able to engage their hip abductors to prevent the prolonged hip adduction seen in the SCI group throughout stance (Fig 1), thus preserving the greater step width.

The narrow base of support experienced by the SCI users may have CNS computational and metabolic energetic cost implications. Previous work has evidenced that step width in able-bodied walking is adjusted to account for head, arm and trunk (HAT) kinematics [36] but that HAT kinematics can also be influenced by step width constraints [37]. Donelan et al. [38] highlighted that reduced sensorimotor information available to the CNS, and associated with lateral stability, led to increased step width variability, which contributed to increased metabolic costs. The SCI group could have used the movement of the trunk as a counterweight to the falling COM. Although the typical response is for the falling COM to be arrested by the placement of the advancing leg, the opposing frontal plane movement pattern observed in the SCI group may have been used to try and maintain the position of the COM inside the advancing base of support. However, this compensation may have further reduced step width, as the lower limbs of the SCI individuals would be more likely to fall towards the midline of the body. Unlike the able-bodied participants, it is possible that this attempt to maintain stability was the most appropriate in terms of CNS computation and was a learned adaptation based on postural control strategies developed during sitting to avoid falling [39]. Although only speculative, because force data could not be collected from the elbow crutches, it is also probable that the SCI users applied greater force through the walking aids compared to the able-bodied group as the medial-lateral force profile under the foot was not different across the groups and core control alone was unlikely to facilitate trunk change of direction in the SCI group. This requirement to use the trunk to control posture may be more energetically costly for the SCI group than for the able-bodied individuals. Additionally, the increased posterior braking force, most likely generated by the motor of the exoskeleton after early ground contact of the anterior limb, led to slower gait speeds and reduced forward progression, but may also pose greater energetic costs for the SCI individual. As such it is suggested that future work should investigate the energetic requirements of overground exoskeleton gait.

This study is the first to present a kinematic analysis of the whole body with COM displacement data, together with GRF data, in overground exoskeleton gait between SCI and able-bodied users. The limited sample size of individuals who had experienced a SCI is one of the primary limitations of this work. However, this group represented approximately 20% of the viable UK population of ReWalk™ active individuals at the time of data collection. There are some other limitations of this study, including the differences in the injury level and severity of the SCI individuals as well as any differences in time since injury which may all have impacted the capacity for the therapeutic effects of the ReWalk™ to activate the latent CPGs. This study was however designed to identify the movement profiles of the different groups rather than to assess the impact these movements may have on the CPGs. Consequently, it is acknowledged that limitations in group homogeneity existed, but that they would not negatively impact the outcome of the study. It is also noted that the SCI participants were asked to complete the walking tasks in the manner they felt most comfortable, safe and competent in relation to the use of crutches. Three of the four participants utilised a four-point gait pattern, advancing one crutch at a time followed by the contralateral leg and one participant used a three-point pattern, advancing both crutches between each step. It is possible that the differences in these movements may have increased the variability in the data between the participants with an SCI. Therefore median, rather than mean, data were used in the analyses, limiting the effect of any variation due to outlying data points.

## Conclusion

The primary findings show that, although some significant differences were evident between the groups, the parameters that would most directly pertain to CPG activation (hip extension and ipsilateral lower limb unloading) presented with no significant between-group differences. These findings suggest that appropriate afferent information was available to elicit a positive response of the CPGs in the lower spinal cord for both able-bodied and SCI users. Therefore, the ReWalk^TM^ can offer potential benefits for SCI individuals with injuries of differing severity. This study was able to identify a link between slower gait speeds, reduced step length yet an increased posterior braking force, which may have energetic cost implications for SCI individuals. Finally, although overground exoskeleton devices use pre-defined movements, the active requirement of the user to balance, facilitate forward movement using the trunk, and the capacity for joint angles to exceed those prescribed by the device suggest that overground exoskeletons produce very similar biomechanical profiles for different user groups. Based on the above findings, the gait patterns for different user groups will be representative of “normal” slow walking; however, the user-device interaction is dependent upon the neuromuscular control of the individual.

## Supporting information

S1 Data (XLSX)

## References

[1] Fliess-Douer O, Vanlandewijck YC, Lubel Manor G, Van Der Woude LH. A systematic review of wheelchair skills tests for manual wheelchair users with a spinal cord injury: towards a standardized outcome measure. Clin Rehabil. 2010 Oct 1;24(10):867–86. doi: 10.1177/0269215510367981 20554638

[2] Minkel JL. Seating and Mobility Considerations for People With Spinal Cord Injury. Phys Ther. 2000 Jul;80(7):701–9. 10869132

[3] Bauman WA, Schwartz E, Song ISY, Kirshblum S, Cirnigliaro C, Morrison N, et al. Dual-energy X-ray absorptiometry overestimates bone mineral density of the lumbar spine in persons with spinal cord injury. Spinal Cord. 2009 Aug;47(8):628–33. doi: 10.1038/sc.2008.169 19153590

[4] Steeves JD, Lammertse D, Curt A, Fawcett JW, Tuszynski MH, Ditunno JF, et al. Guidelines for the conduct of clinical trials for spinal cord injury (SCI) as developed by the ICCP panel: clinical trial outcome measures. Spinal Cord. 2007 Mar;45(3):206–21. doi: 10.1038/sj.sc.3102008 17179972

[5] Masani K, Sin VW, Vette AH, Thrasher TA, Kawashima N, Morris A, et al. Postural reactions of the trunk muscles to multi-directional perturbations in sitting. Clin Biomech Bristol Avon. 2009 Feb;24(2):176–82. doi: 10.1016/j.clinbiomech.2008.12.001 19150744

[6] Alekna V, Tamulaitiene M, Sinevicius T, Juocevicius A. Effect of weight-bearing activities on bone mineral density in spinal cord injured patients during the period of the first two years. Spinal Cord. 2008 Nov;46(11):727–32. doi: 10.1038/sc.2008.36 18443599

[7] Harvey LA, Herbert RD. Muscle stretching for treatment and prevention of contracture in people with spinal cord injury. Spinal Cord. 2002 Jan;40(1):1–9. doi: 10.1038/sj.sc.3101241 11821963

[8] Hicks AL, Martin Ginis KA, Pelletier CA, Ditor DS, Foulon B, Wolfe DL. The effects of exercise training on physical capacity, strength, body composition and functional performance among adults with spinal cord injury: a systematic review. Spinal Cord. 2011 Nov;49(11):1103–27. doi: 10.1038/sc.2011.62 21647163

[9] Hubli M, Dietz V. The physiological basis of neurorehabilitation—locomotor training after spinal cord injury. J NeuroEngineering Rehabil. 2013 Jan;10(1):5. doi: 10.1186/1743-0003-10-5 23336934 PMC3584845

[10] Mikolajewska E, Mikolajewski D. Exoskeletons in Neurological Diseases—Current and Potential Future Applications. Adv Clin Exp Med. 2011;20(2):227–33.

[11] Ramanujam A, Cirnigliaro CM., Garbarini E, Asselin P, Pilkar R, Forrest GF. Neuromechanical adaptations during a robotic powered exoskeleton assisted walking session. J Spinal Cord Med. 2017;41(5):518–28. doi: 10.1080/10790268.2017.1314900 28427305 PMC6117573

[12] Louie DR, Eng JJ, Lam T. Gait speed using powered robotic exoskeletons after spinal cord injury: a systematic review and correlational study. J Neuroengineering Rehabil. 2015 Oct;12:82. doi: 10.1186/s12984-015-0074-9 26463355 PMC4604762

[13] Viteckova S, Kutilek P, Jirina M. Wearable lower limb robotics: A review. Biocybern Biomed Eng. 2013 Jan 1;33(2):96–105.

[14] Hayes SC, Wilcox CRJ, White HSF, Vanicek N. The effects of robot assisted gait training on temporal-spatial characteristics of people with spinal cord injuries: A systematic review. J Spinal Cord Med. 2018 Sep 3; 41(5):529–43. doi: 10.1080/10790268.2018.1426236 29400988 PMC6117598

[15] Minassian K, Hofstoetter US, Dzeladini F, Guertin PA, Ijspeert A. The Human Central Pattern Generator for Locomotion: Does It Exist and Contribute to Walking? The Neuroscientist. 2017 Dec 1;23(6):649–63. doi: 10.1177/1073858417699790 28351197

[16] Field-Fote EC, Lindley SD, Sherman AL. Locomotor training approaches for individuals with spinal cord injury: a preliminary report of walking-related outcomes. J Neurol Phys Ther JNPT. 2005 Sep;29(3):127–37. doi: 10.1097/01.npt.0000282245.31158.09 16398945

[17] Hornby G, Straube D, Kinnaird C, Holleran C, Echauz A, Rodriguez K, et al. Importance of Specificity, Amount, and Intensity of Locomotor Training to Improve Ambulatory Function in Patients Poststroke. Top Stroke Rehabil. 2011 Jul;18(4):293–307. doi: 10.1310/tsr1804-293 21914594

[18] Levin MF, Weiss PL, Keshner EA. Emergence of virtual reality as a tool for upper limb rehabilitation: incorporation of motor control and motor learning principles. Phys Ther. 2015 Mar;95(3):415–25. doi: 10.2522/ptj.20130579 25212522 PMC4348716

[19] Hayes SC, White M, White HSF, Vanicek N. A biomechanical comparison of powered robotic exoskeleton gait with normal and slow walking: An investigation with able-bodied individuals. Clin Biomech. 2020 Dec 1;80:105133. doi: 10.1016/j.clinbiomech.2020.105133 32777685

[20] Horak FB. Postural orientation and equilibrium: what do we need to know about neural control of balance to prevent falls? Age Ageing. 2006 Sep;35 Suppl 2(suppl_2):ii7–11. doi: 10.1093/ageing/afl077 16926210

[21] Winter DA, Patla AE, Frank JS. Assessment of balance control in humans. Med Prog Technol. 1990 May;16(1–2):31–51. 2138696

[22] ReWalk. ReWalk Certification Course: Basic Clinical Training [Course Manual]. 2014.

[23] Cappozzo A, Catani F, Della Croce U, Leardini A. Position and orientation in space of bones during movement: anatomical frame definition and determination. Clin Biomech. 1995;10(4):171–8. doi: 10.1016/0268-0033(95)91394-t 11415549

[24] Buczek FL, Rainbow MJ, Cooney KM, Walker MR, Sanders JO. Implications of using hierarchical and six degree-of-freedom models for normal gait analyses. Gait Posture. 2010 Jan;31(1):57–63. doi: 10.1016/j.gaitpost.2009.08.245 19796947

[25] Dunn OJ. Multiple Comparisons Using Rank Sums. Technometrics. 1964;6(3):241–52.

[26] Cliff N. Ordinal Methods for Behavioral Data Analysis [Internet]. Psychology Press; 2014 [cited 2020 Feb 21]. Available from: https://www.taylorfrancis.com/books/9781315806730.

[27] Romano J, Kromery JD, Coraggio J, Skowronek J, Devine L. Exploring methods for evaluating group differences on the NSSE and other surveys: are the t-test and Cohen’s d indices the most appropriate choices. In Arlington, VA,; 2006.

[28] Zeilig G, Weingarden H, Zwecker M, Dudkiewicz I, Bloch A, Esquenazi A. Safety and tolerance of the ReWalkTM exoskeleton suit for ambulation by people with complete spinal cord injury: a pilot study. J Spinal Cord Med. 2012 Mar;35(2):96–101. doi: 10.1179/2045772312Y.0000000003 22333043 PMC3304563

[29] Hidler J, Wisman W, Neckel N. Kinematic trajectories while walking within the Lokomat robotic gait-orthosis. Clin Biomech. 2008;23(10):1251–9. doi: 10.1016/j.clinbiomech.2008.08.004 18849098

[30] Reier PJ, Howland DR, Mitchell G, Wolpaw JR, Hoh D, Lane MA. Spinal Cord Injury: Repair, Plasticity and Rehabilitation. 2017; Available from: www.els.net.

[31] Cai LL, Fong AJ, Otoshi CK, Liang YQ, Cham JG, Zhong H, et al. Effects of consistency vs. variability in robotically controlled training of stepping in adult spinal mice. In: 9th International Conference on Rehabilitation Robotics, 2005 ICORR 2005. 2005. p. 575–9.

[32] Labruyère R, van Hedel HJA. Strength training versus robot-assisted gait training after incomplete spinal cord injury: a randomized pilot study in patients depending on walking assistance. J NeuroEngineering Rehabil. 2014;11(1):4. doi: 10.1186/1743-0003-11-4 24401143 PMC3905290

[33] Lam T, Pauhl K, Krassioukov A, Eng JJ. Using Robot-Applied Resistance to Augment Body-Weight–Supported Treadmill Training in an Individual With Incomplete Spinal Cord Injury. Phys Ther. 2011 Jan;91(1):143–51. doi: 10.2522/ptj.20100026 21127165

[34] Nooijen CFJ, ter Hoeve N, Field-Fote EC. Gait quality is improved by locomotor training in individuals with SCI regardless of training approach. J NeuroEngineering Rehabil. 2009;6(1):36. doi: 10.1186/1743-0003-6-36 19799783 PMC2764722

[35] Rosenblatt NJ, Grabiner MD. Measures of frontal plane stability during treadmill and overground walking. Gait Posture. 2010 Mar;31(3):380–4. doi: 10.1016/j.gaitpost.2010.01.002 20129786

[36] Hurt CP, Rosenblatt N, Crenshaw JR, Grabiner MD. Variation in trunk kinematics influences variation in step width during treadmill walking by older and younger adults. Gait Posture. 2010 Apr;31(4):461–4. doi: 10.1016/j.gaitpost.2010.02.001 20185314

[37] Arvin M, Mazaheri M, Hoozemans MJM, Pijnappels M, Burger BJ, Verschueren SMP, et al. Effects of narrow base gait on mediolateral balance control in young and older adults. J Biomech. 2016 May;49(7):1264–7. doi: 10.1016/j.jbiomech.2016.03.011 27018156

[38] Donelan JM, Shipman DW, Kram R, Kuo AD. Mechanical and metabolic requirements for active lateral stabilization in human walking. J Biomech. 2004 Jun;37(6):827–35. doi: 10.1016/j.jbiomech.2003.06.002 15111070

[39] Chisholm AE, Alamro RA, Williams AMM, Lam T. Overground vs. treadmill-based robotic gait training to improve seated balance in people with motor-complete spinal cord injury: a case report. J NeuroEngineering Rehabil. 2017 Dec;14(1):27. doi: 10.1186/s12984-017-0236-z 28399877 PMC5387335

